# Comprehensive construction strategy of bidirectional green tissue‐specific synthetic promoters

**DOI:** 10.1111/pbi.13231

**Published:** 2019-08-19

**Authors:** Jiuyuan Bai, Xin Wang, Hao Wu, Fei Ling, Yun Zhao, Yongjun Lin, Rui Wang

**Affiliations:** ^1^ Key Laboratory of Bio‐Resource and Eco‐Environment of Ministry of Education College of life sciences Sichuan University Chengdu China; ^2^ National Key Laboratory of Crop Genetic Improvement and National Centre of Plant Gene Research Huazhong Agricultural University Wuhan China

**Keywords:** synthetic promoters, tissue‐specific expression, bidirectional expression, *cis*‐elements, genetic engineering

## Abstract

Bidirectional green tissue‐specific promoters have important application prospects in genetic engineering and crop genetic improvement. However, there is no report on the application of them, mainly due to undiscovered natural bidirectional green tissue‐specific promoters and the lack of a comprehensive approach for the synthesis of these promoters. In order to compensate for this vacancy, the present study reports a novel strategy for the expression regulatory sequence selection and the bidirectional green tissue‐specific synthetic promoter construction. Based on this strategy, seven promoters were synthesized and introduced into rice by *agrobacterium*‐mediated transformation. The functional identification of these synthetic promoters was performed by the expression pattern of *GFP* and *GUS* reporter genes in two reverse directions in transgenic rice. The results indicated that all the synthetic promoters possessed bidirectional expression activities in transgenic rice, and four synthetic promoters (*BiGSSP2*,* BiGSSP3*,* BiGSSP6*,* BiGSSP7*) showed highly bidirectional expression efficiencies specifically in green tissues (leaf, sheath, panicle, stem), which could be widely applied to agricultural biotechnology. Our study provided a feasible strategy for the construction of synthetic promoters, and we successfully created four bidirectional green tissue‐specific synthetic promoters. It is the first report on bidirectional green tissue‐specific promoters that could be efficiently applied in genetic engineering.

## Introduction

The morphogenesis, development and environmental adaptation of plants are closely related to the spatio‐temporal expression of specific genes (Chen *et al*., 2010b; Mcelroy *et al*., 1990; Zhu *et al*., 2011). In the regulatory system of gene expression, the promoter, serving as an important regulatory element at the transcriptional level, controls the transcription initiation and expression abundance of downstream gene by interacting with different *trans‐*acting factors (Balasubramani *et al*., 2014; Walcher and Nemhauser, 2012; Yi *et al*., 2011), which makes the study on promoters possess a great application potential and theoretical value (Chen *et al*., 2016; Mcelroy *et al*., 1990; Pan *et al*., 2015; Vijayan *et al*., 2015).

Synthetic biology, a discipline to build new biological components and systems combining biological science and engineering or to redesign natural biological systems, has shown great potential in many fields including promoter synthesis (Baltes and Voytas, 2015; Mauritz, 2007). Compared with the native promoter, synthetic promoter allows for more flexible assembly of the function‐known regulatory sequences based on different purposes to improve the expression accuracy of genes under specific time, space and signal stimuli (Li *et al*., 2013). At present, studies on the construction of synthetic promoters mainly involve in microbial synthetic promoters. For the purpose of screening expected promoters, fusion promoters are usually constructed by appending large quantities of *cis*‐regulatory elements or random sequences to the core promoter, and these fusion promoters are subsequently transformed into different bacteria or fungi to detect the expression patterns and abundance of the reporter gene (Jakob Vang *et al*., 2014; Sohoni *et al*., 2014). However, this strategy is not suitable for plant synthetic promoters because of the long transformation cycle and the vast identification procedures. Therefore, the reports on plant synthetic promoter are relatively few and confined to inducible synthetic promoter. For example, Li *et al*. (2013) designed and identified a plant defence signalling molecules‐related inducible synthetic promoter in transgenic tobacco and *Arabidopsis thaliana*. The results showed that the reporter genes expressed after suffering from pathogens, salicylic acid, ethylene and jasmonate acid treatment.

Tissue‐specific promoters, referring generally to driving gene expression in a specific tissue or organ, are widely concerned in genetic engineering, such as green tissue‐specific promoter (Molla *et al*., 2013), endosperm‐specific promoter (Ha *et al*., 2010) and root‐specific expression promoter (Ahn *et al*., 2014). However, few researches of tissue‐specific synthetic promoters in plant have been reported mainly for the reason that the method for promoter construction by single *cis*‐element fusing with a core promoter is hard to meet the two demands to promote the expression of exogenous gene in target tissue and simultaneously inhibit its expression in non‐target tissues. Besides, the reports on the construction of tissue‐specific synthetic promoters by fusing multiple *cis*‐elements are also rare on account of the difficulty in determining the arrangement of multiple *cis*‐elements.

As the development of crop genetic improvement and functional genomics research, crop traits such as stress resistance, nutrient utilization, yield and quality are attracting more and more attention. Simultaneously, several lines of candidate genes for crop trait improvement emerged, and their effective application depends on their efficient expression at specific sites, growth stages or signal stimulation (Wang *et al*., 2016). Therefore, the diversity of promoters is urgently needed in genetic engineering. However, the development and application of expression regulatory elements in genetic engineering are mostly limited to unidirectional promoters and barely involved in bidirectional promoters. Bidirectional promoters are referred to the sequence between pairs of genes that are adjacent and oriented in head‐to‐head way (Mitra *et al*., 2009; Song *et al*., 2013; Trinklein *et al*., 2004), as a result, these promoters possess wider application than unidirectional promoters in driving the expression of two target genes simultaneously. Besides, bidirectional promoters also provide great convenience for vector construction and gene aggregation (Kumar *et al*., 2015). On the other hand, the improvement of a specific trait by expressing multiple exogenous genes is commonly demanded to maintain the same or similar expression pattern (Ogo *et al*., 2013). However, the number of unidirectional promoters with the same or similar expression patterns is limited, and the repeated use of these promoters *in vivo* may cause silencing of gene expression (Chen *et al*., 2010b; Wang *et al*., 2009). These obstacles could be overcome by bidirectional promoters based on the quite similar expression pattern in both 3′ and 5′ directions (Didych *et al*., 2013). With the improvement of high‐throughput gene expression databases such as expression profiling and transcriptome sequencing data, a cascade of reports on cloning and identification of unidirectional promoters were presented. Nevertheless, the exploration and application process of the bidirectional promoter is quite slow, which could be attributed to the lack of a highly efficient approach for screening and construction of bidirectional promoter.

Here, in order to meet the extensive demands of genetic engineering, we designed a novel comprehensive strategy for the selection and assembly of expression regulatory sequences. Subsequently, several bidirectional green tissue‐specific synthetic promoters were constructed from the selected expression regulatory sequences based on our strategy. These synthetic promoters were identified by determining the expression pattern and abundance of reporter genes (*GFP* and *GUS*) simultaneously in two divergent directions in transgenic rice. The results indicated that four synthetic bidirectional promoters (*BiGSSP2*,* BiGSSP3*,* BiGSSP6* and *BiGSSP7*) showed highly bidirectional expression efficiencies specifically in green tissues (leaf, sheath, panicle and stem). In conclusion, we provided a feasible strategy for the construction of bidirectional tissue‐specific synthetic promoter, and we successfully constructed and identified four bidirectional green tissue‐specific synthetic promoters, which is the first report on bidirectional green tissue‐specific promoters and provided significant promoters resources for genetic engineering.

## Results

### The activities of synthetic promoters compared with *PD* and *RB*

**Figure 1:**
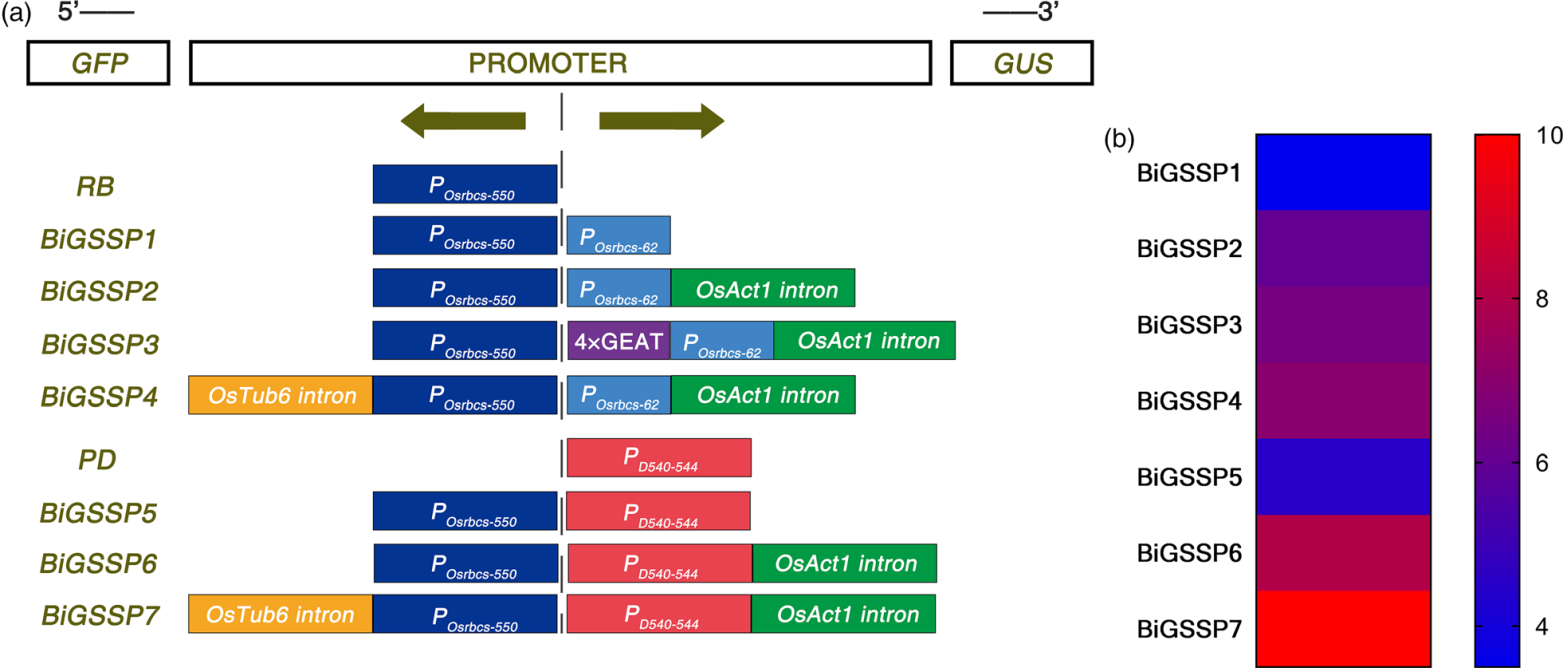
The synthetic promoter assembly and scoring. (a) Schematic diagram of synthetic promoters with different expression regulatory sequences. The position of promoters and reporter genes on the vector is shown at the top of the diagram. The bold arrows indicate the transcription directions of promoters. The left side of the diagram shows the name of synthetic promoters. Different coloured rectangles represent different expression regulation sequences. (b) Comprehensive score of synthetic promoter based on scoring rules. The different colour represents different score.

The synthetic promoters were designed and constructed based on our assembling strategy shown in Figure 1, Tables S1 and S2. The unidirectional promoter (i.e. *P*_*Osrbcs‐550*_, *P*_*D540‐544*_), namely *RB* and *PD* in the present study, respectively, were used as control. For the qualitative and quantitative analysis of promoter expression efficiency in two reverse directions, the reporter genes *GUS* and *GFP* were located in the 3′ and 5′ orientations, respectively, which closely adjoined to the promoter region.

**Figure 2:**
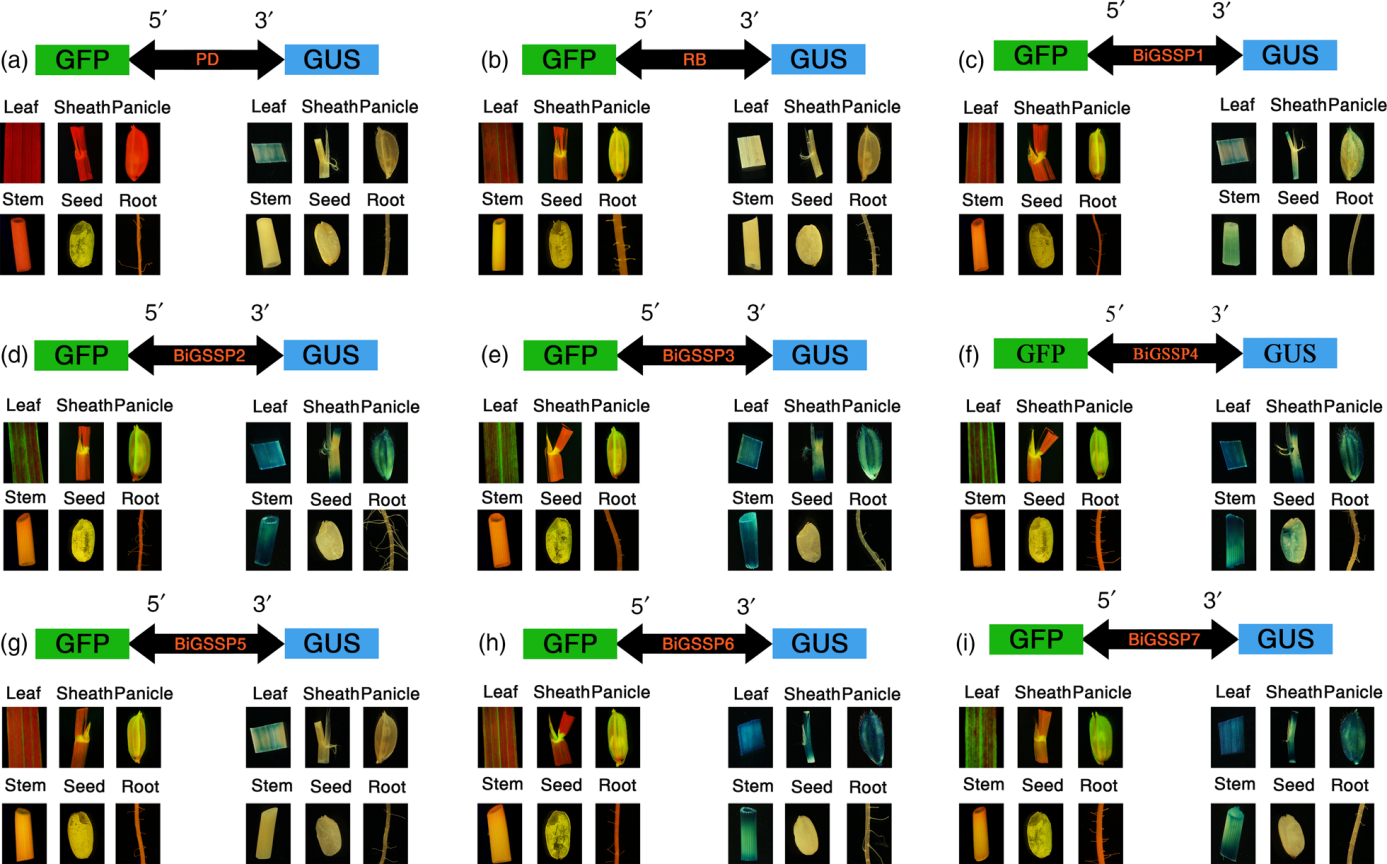
Histological analysis of GFP and GUS expression in various tissues of the transgenic plants containing different *GFP*/synthetic promoter/*GUS* fusions. (a) *PD*; (b) *RB*; (c‐i) *BiGSSP1*‐*BiGSSP7*.

By means of GUS and GFP assays of the transgenic plants, explicit bidirectional and green tissue‐specific expression pattern can be observed in all synthetic promoters constructed herein from the result of GUS histochemical stain and GFP fluorescence intensity, except the expression of *GUS* in endosperm of *BiGSSP4* (Figure 2). In order to quantitatively analyse the expression of *GUS* and *GFP* in diverse synthetic promoters, GUS enzymatic activity and *GFP* relative expression level (standardized by the expression level in stem of *RB*) are measured.

**Figure 3:**
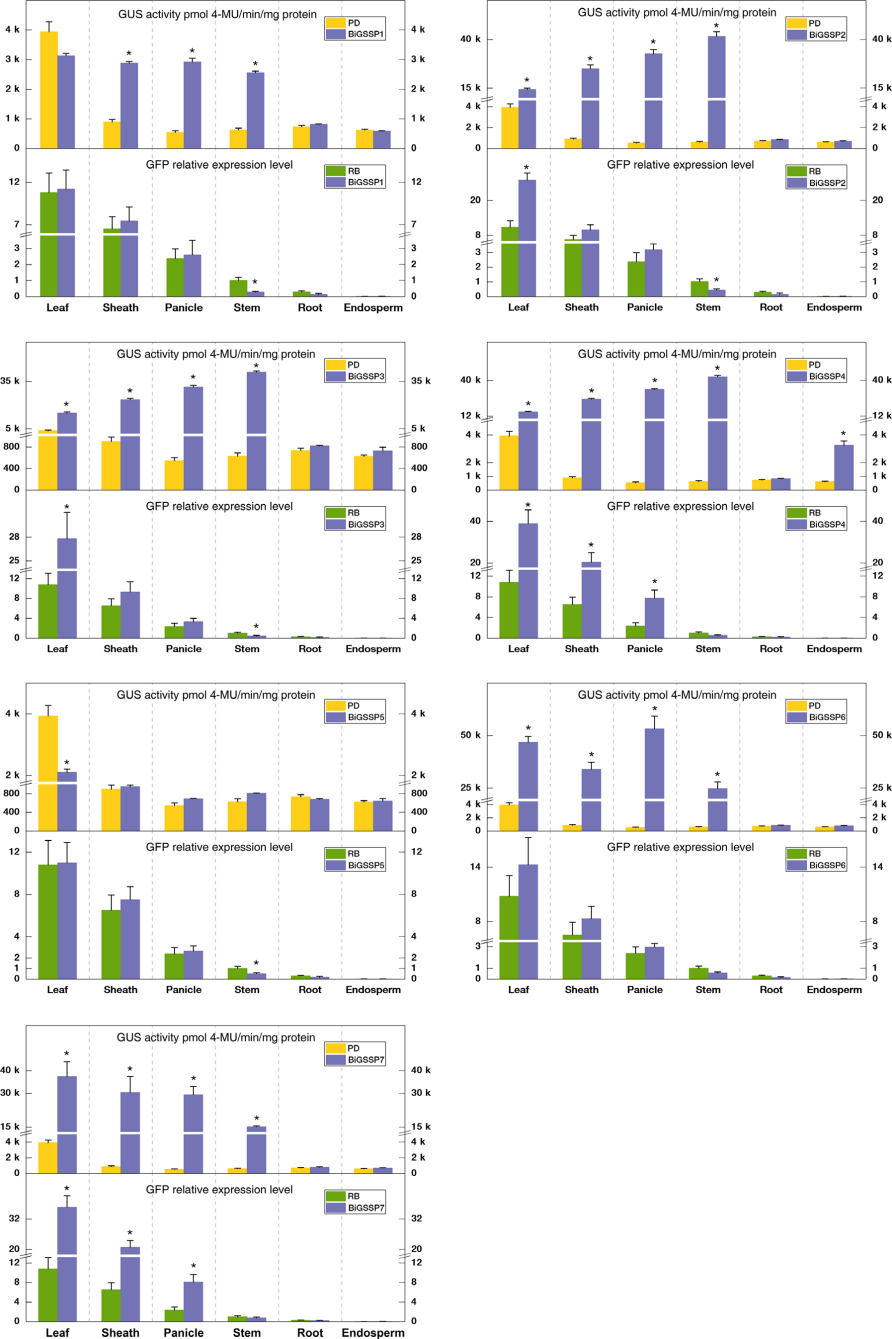
Quantitative analysis of *GFP* and *GUS* expression in various tissues of the transgenic plants. The GFP relative expression level was standardized by the GFP expression in stem of *RB*. Significance analysis was conducted by independent‐samples *t*‐test. * represents *P* < 0.05; ** represents *P* < 0.01. Error bars indicate standard error based on three independent biological replicates.

Quantitative results of expression efficiency concerning each synthetic promoter in 3′ (*GUS*) and 5′ (*GFP*) directions in comparison with the corresponding control are shown in Figure 3. Regarding the controls alone, the enzymatic activity of GUS promoted by control *PD* reaches up to 3939 ± 337 pmol 4‐MU/min/mg protein in leaves in sharp contrast to the scarce activity in other tissues (<1000 pmol 4‐MU/min/mg protein). Thereby, it can be manifested that *PD* itself has apparent green tissue specificity (even leaf specificity). Similarly, single promoter *RB* shows certain green tissue specificity. The highest expression level of *GFP* appears in leaf among six tissues, followed by in sheath and almost no expression in root and endosperm, which is inconsistent with the previous research (Huang, 2006). From a holistic perspective, all of the bidirectional synthetic promoters constructed in the present experiment showed rare expression efficiency in root and endosperm reflecting significant green tissue expression specificity (except *BIGSSP4*), whether for *GFP* or *GUS* expression, which can be verified along with Figure 3. Moreover, the expression of *GFP* in all synthetic promoter transgenic plants exhibits relatively low level in stem, which can be explained by the previous result that *P*_*Osrbcs‐550*_ inhibits the gene expression in stem when integrated in synthetic promoter (Wang *et al*., 2015b).

The expression regulatory sequence of *BiGSSP1* in the *GUS* direction is *P*_*Osrbcs‐62*_, which is truncated from *P*_*Osrbcs‐550*_ (Huang, 2006). In spite of the GUS activity of *BiGSSP1* in leaf was slightly lower than *PD*, the GUS activity in other green tissues is uniformly higher than *PD* and reaches 2881 ± 58, 2923 ± 120 and 2558 ± 52 pmol 4‐MU/min/mg protein in sheath, panicle and stem, respectively. However, no significant difference in the *GFP* expression driven by *BiGSSP1* and *PD* can be found due to the identical regulatory sequence in 5′ orientation in the two promoters. In the 3′ direction of *BiGSSP2*, an *OsAct1* is added downstream the *P*_*Osrbcs‐62*_. It can be seen that GUS activity of *BiGSSP2* transgenic rice is greatly improved compared with control, which is 3.6, 27.8, 59.7 and 65.9 times that of *PD* transgenic rice in leaf, sheath, panicle and stem, respectively. With respect to *GFP* expression, although the expression regulatory sequences of *BiGSSP2* and *BiGSSP1* in the *GFP* direction are identical with the control *RB*, the expression activity of *BiGSSP2* in leaf is 2.5‐fold than that of *RB*, while other green tissues perform similar expression levels. This phenomenon can be attributed to the significant enhancement to *P*_*Osrbcs‐550*_ in leaf by the upstream reverse *OsAct1* in *BiGSSP2* (Figure 1). Synthetic promoter *BiGSSP3* was constructed by adding four tandem GEAT *cis*‐elements in the upstream of *P*_*Osrbcs‐62*_ in *BiGSSP2*. Nevertheless, no distinct positive effect arises in line with our expectation. Both GUS activity and *GFP* expression level are similar to *BiGSSP2* which is 2.57‐fold *GFP* expression level in leaf than *RB*. The activity of GUS in *BiGSSP4* is also considerably increased by contrast to *PD*, which exhibits similar expression pattern with *BiGSSP2* and *BiGSSP3*, and reaches 15 284 ± 454, 25 336 ± 586, 33 038 ± 443 and 42 778 ± 1212 pmol 4‐MU/min/mg protein in leaf, sheath, panicle and stem. Surprisingly, given all the synthetic promoters constructed herein, only *BiGSSP4* showed relatively high GUS activity in the endosperm (2321 ± 279 pmol 4‐MU/min/mg protein). With an *OsTub6I* subjoined to the downstream of *BiGSSP4* at the *GFP* direction based on *BiGSSP3*, the expression level of *GFP* in all green tissues (except stem) shows strong improvement than *BiGSSP3* and is 3.6‐, 3.1‐ and 3.3‐fold than control *RB* in leaf, sheath and panicle.

*BiGSSP5* is a synthetic bidirectional promoter constituted by corresponding control *P*_*Osrbcs‐550*_ and *P*_*D540‐544*_ alone at the 5′ and 3′ directions, respectively. Unsurprisingly, the expression of *GFP* and *GUS* in both directions are similar to the respective control while the GUS activity in the leaf become an exception, which is only 2108 ± 103 pmol 4‐MU/min/mg protein and is significantly lower (54.0%) than *PD*. The promoter of *BiGSSP6* in the 5′ direction is also identical to *RB*, and similarly, parallel expression efficiency is exhibited. However, the *OsAct1* downstream of the *P*_*D540‐544*_ causes a huge difference in GUS activity than *PD*, which is significantly increased to 10.9, 36.8, 96.5 and 38.4 times in leaf, sheath, panicle and stem, respectively, but not in non‐green tissues. *BiGSSP7* based on *BiGSSP6* harbours an *OsTub6I* linked downstream of the *P*_*Osrbcs‐550*_ towards the 5′ direction, and the regulatory sequence at 3′ direction is the same as *BiGSSP6*. Expectedly, like *BiGSSP6*, the GUS activity is also significantly improved in all four green tissues (8.5‐, 32.8‐, 52.7‐ and 23.2‐folds, respectively) in contrast to *PD* (but slightly weaker than *BiGSSP6*). Similar to *BiGSSP4*, the *GFP* expression level is also raised substantially in leaf, sheath and panicle versus *RB* on account for the positive role brought by downstream *Ostub6 intron*.

### Intercomparison between the activities of synthetic promoters and *35S* promoter

**Figure 4:**
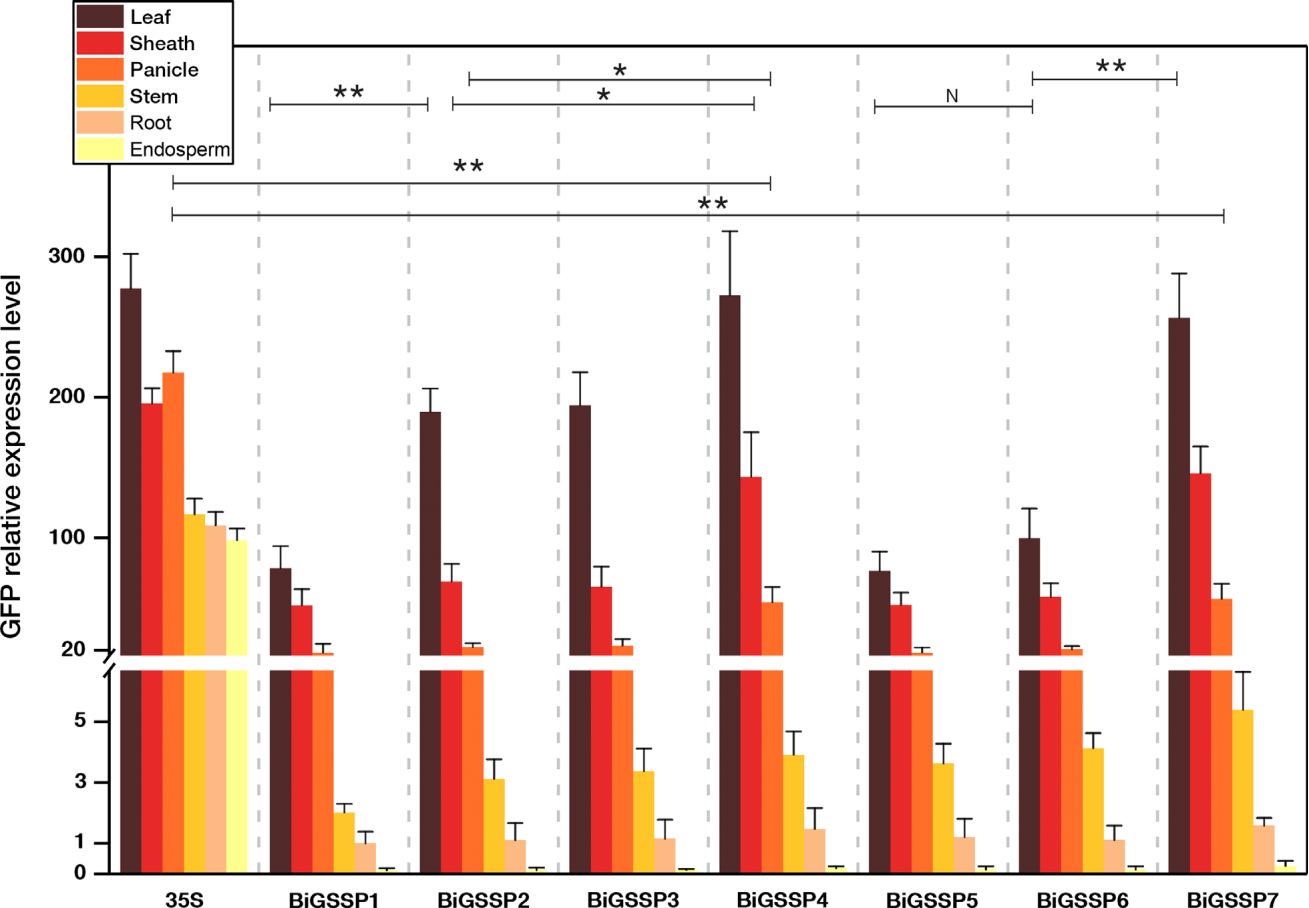
Quantitative analysis of *GFP* expression in various tissues of the transgenic plants. The *GFP* relative expression level was standardized by the *GFP* expression in root of *BiGSSP1*. Significance analysis was conducted by independent‐samples *t*‐test. * represents *P* < 0.05; **represents *P* < 0.01; N represents no significant difference (*P* > 0.05). Error bars indicate standard error based on three independent biological replicates.

**Figure 5:**
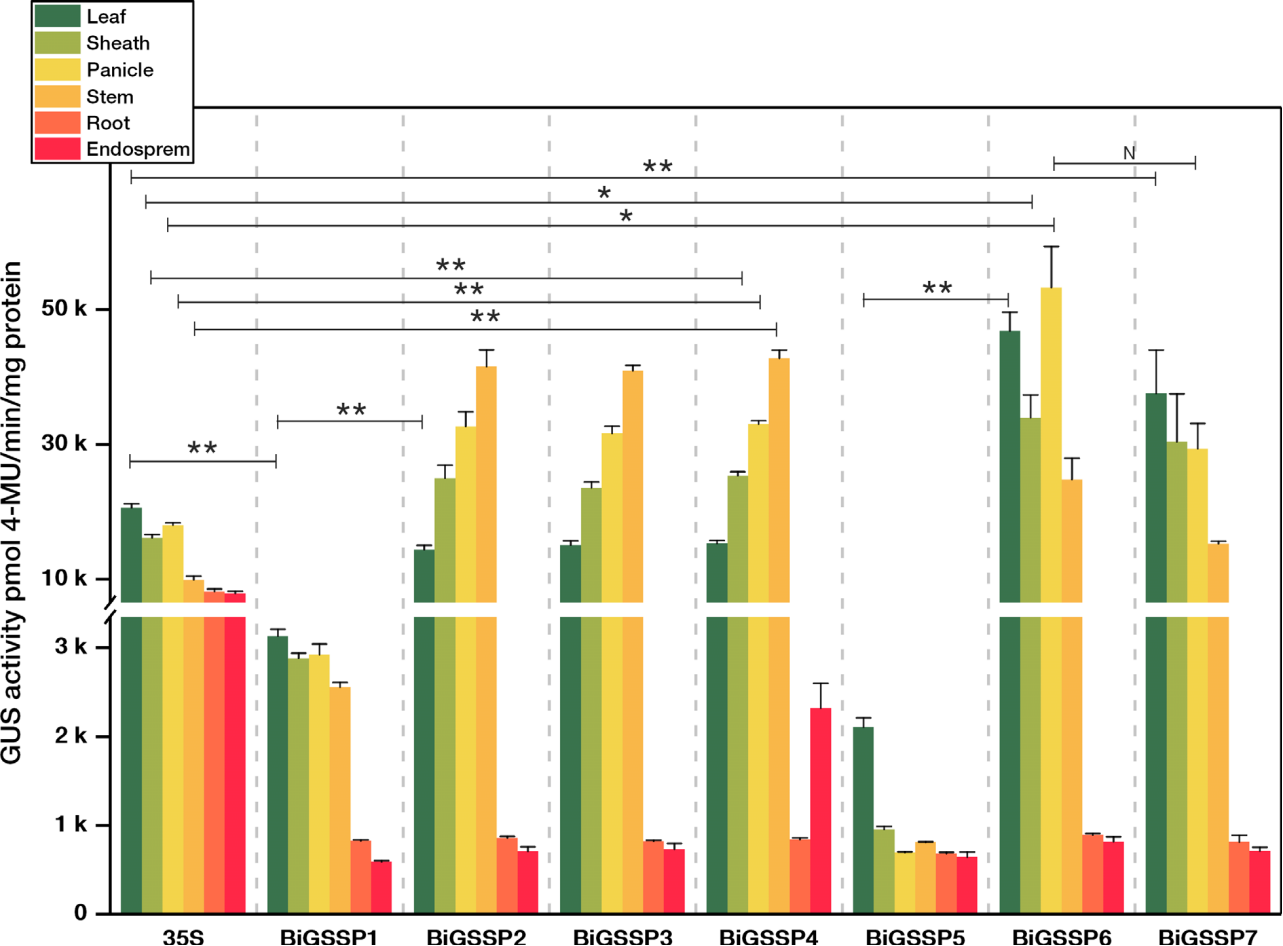
Quantitative analysis of GUS expression in various tissues of the transgenic plants. Significance analysis was conducted by independent‐samples *t*‐test. * represents *P* < 0.05; **represents *P* < 0.01; *N* represents no significant difference (*P* > 0.05). Error bars indicate standard error based on three independent biological replicates.

With the intention to intuitively compare the expression levels of each constructed synthetic promoters along with the regulatory effect of the corresponding promoters and elements in synthetic promoter, we integrally compared the expression intensities of the different promoters in Figures 4 and 5. Moreover, the application potential is particularly important in plant genetic improvement. To this end, the constitutive promoter CaMV *35S* is selected, which is very commonly used in practice, as a comparison object.

Figure 4 shows the relative expression level of *GFP* (standardized by the expression level in root of *BiGSSP1*) at the 5′ direction of each synthetic promoter in various tissues of rice. Obvious green tissue specificity can be found in accordance with Figure 2, while *35S* promoter exhibits high activity in all tissues. The intercomparison of each synthetic promoter indicates that the order of *GFP* expression efficiency in green tissues generally following the pattern that 35S≈BiGSSP4≈BiGSSP7>BiGSSP2≈ BiGSSP3>BiGSSP6≈BiGSSP5≈BiGSSP1. Compared with *BiGSSP1*,* BiGSSP2* and *BiGSSP3* increases, the *GFP* expression level in leaf by about 2.5‐fold, indicating the significant positive effect brought by upstream reverse *OsAct1* in leaf but not in any other tissues. However, there is no significant disagreement between *BiGSSP2* and *BiGSSP3* in the expression of *GFP*, indicating that the upstream reverse 4×GEAT *cis*‐element carries no regulatory effect on the expression efficiency. Interestingly, unlike *BiGSSP2*, the inverted *OsAct1* in *BiGSSP6* only increases the expression of *GFP* in leaf by 30.1% with no significant difference compared with *BiGSSP5*, where the enhancement effect is greatly reduced than that in *BiGSSP2*. Given the much shorter sequence of *P*_*Osrbcs‐62*_ than *P*_*D540‐544*_, it can be deduced that the length of the interval sequence between *P*_*Osrbcs‐550*_ and the upstream reverse *OsAct1* plays a key role on the regulatory effect of *OsAct1*. Both *BiGSSP4* and *BiGSSP7* possess similarly high *GFP* expression efficiency. In leaf, sheath and panicle, the expression efficiencies of *BiGSSP4* is 1.44‐, 2.08‐ and 2.42‐fold higher than *BiGSSP2*, and that of *BiGSSP7* is 2.57‐, 2.51‐ and 2.74‐fold higher than *BiGSSP6*. This further confirms the strong and non‐tissue‐specific promoting effect of *Ostub6 intron* in several green tissues. Overall, *BiGSSP4* and *BiGSSP7* showed relatively higher expression efficiency in 5′ direction among all synthetic promoters in the present study. The expression efficiency could represent the application potential to certain extent when compared with *35S* promoter. As for green tissues in rice, the expression efficiency of *GFP* in *BiGSSP4* and *BiGSSP7* is extremely lower than *35S* promoter in panicle and stem. However, similar expression efficiency in leaf and sheath compared with *35S* still indicates the high value for application to some degree.

The intercomparison of GUS enzymatic activity between each synthetic promoter and *35S* promoter in various green tissues of rice is shown in Figure 5. Overall, although all synthetic promoters perform high green tissue specificity, the expression pattern of *GUS* in each synthetic promoter shows big divergences in green tissues. The GUS activity of the *35S* promoter is highly expressed in all tissues, and the highest expression level occurs in leaves (20 584 ± 558 pmol 4‐MU/min/mg protein). The GUS activity of *BiGSSP1* is very low in each green tissue (<3100 pmol 4‐MU/min/mg protein), while *BiGSSP5* is almost only expressed in leaf with little expression in sheath, panicle and stem. The GUS activity of *BiGSSP1* and *BiGSSP5* in leaf is significantly lower than *35S*, which is only 15.0% and 10.0% of *35S,* respectively. The GUS activities of *BiGSSP2*,* BiGSSP3* and *BiGSSP4* at the 3′ direction are very coincident. Their GUS activities are all significantly higher than that of the *35S* promoter in green tissues (except leaf), especially highest in stem, where the GUS activity is threefold higher than *35S*, followed by panicle, sheath and leaf. We have testified that the inhibition of activity in stem existing in *P*_*Osrbcs‐550*_ is abolished in *P*_*Osrbcs‐62*_ in former research (Wang *et al*., 2015b). Hence, unlike *GFP* in 5′ direction, the high expression efficiency of *GUS* in stem may be attributed to the revocatory inhibition in *P*_*Osrbcs‐62*_. Compared with *BiGSSP1*,* BiGSSP2* shows increased GUS activity in leaf, sheath, panicle and stem by 3.6, 7.7, 10.2 and 15.2 times, respectively, while *BiGSSP3* and *BiGSSP4* exhibit much the same expression efficiency as *BiGSSP2*, which indicates that the addition of *OsAct1* downstream can greatly promote the activity of *P*_*Osrbcs‐62*_ in green tissues, especially in stem. And the addition of 4×GEAT *cis*‐element (*BiGSSP3*) or reversed *OsTub6I* (*BiGSSP4*) upstream *P*_*Osrbcs‐62*_ has no effect on the expression efficiency of GUS, compared with *BiGSSP2*. However, it is unexpected that the only GUS activity in endosperm appears in *BiGSSP4*, which may be attributed to the interaction between *Ostub6 intron* and *P*_*Osrbcs‐62*_. With regard to *BiGSSP6* and *BiGSSP7*, the GUS activities are both significantly higher than *35S* promoter in diverse green tissues, which suggests a huge application potential in genetic engineering. Among all promoters, the highest GUS activity in leaf and panicle both appears in *BiGSSP6*, 2.3‐fold and 3.0‐fold higher, respectively, compared with the *35S* promoter. The increased activity of *BiGSSP6* and *BiGSSP7* compared with *BiGSSP5* confirms the previous research that the coexistence of *P*_*D540‐544*_ and *OsAct1* can markedly increase the promoter expression efficiency in panicle (Wang *et al*., 2015b). In total, *BiGSSP2*,* BiGSSP3* and *BiGSSP4* express the highest GUS activity in stem, while the activity of *BiGSSP6* is the highest among all synthetic promoters both in leaf, sheath and panicle. In addition to the synthetic promoters of *BIGSSP1*,* BIGSSP4* and *BiGSSP5*, all other synthetic promoters constructed in this experiment exhibit strong green tissue specificity and expression efficiency (similar or higher than 35S) in 3′ direction, which indicates a strong application potential.

## Discussion

Upon a variety of purposes in plant genetic engineering, the demand for more precise and diverse gene expression patterns will continue to accelerate the process for seeking or artificially synthesizing more abundant promoters. The construction and application of synthetic promoters increasingly become an effective approach in genetic engineering. Tissue‐specific promoters can provide more precise control of gene expression in certain tissues leaving other tissues unaffected. The construction and application of synthetic promoters is a very effective approach in genetic engineering. In some cases, when multiple foreign genes are used in transgene process, it is often necessary to maintain the similar or diverse expression patterns for the purpose of improving certain traits (Mitra *et al*., 2009; Ogo *et al*., 2013). However, the number of native promoter with same or similar expression patterns is limited, and the repeat utilization may result in silencing of gene expression leading to the excess consumption of time and money (Peremarti *et al*., 2010). To this end, the synthesis of bidirectional promoters becomes an adequate method. In the present study, through certain strategy for screening optimal combinations of various expression regulatory sequences, we initially and successfully constructed four high efficient bidirectional green tissue‐specific synthetic promoters.

To arrange the expression regulatory sequences is critical for constructing synthetic promoters, while the arrangement of expression regulatory sequences is also difficult because different arrangements produce diverse expression patterns and efficiencies (Mauritz, 2007; Puente *et al*., 1996; Wang *et al*., 2015b). The regulatory sequences and *cis*‐acting elements selected in this experiment are all related to green tissue specificity. Consequently, all synthetic promoters remained this specificity expect *BiGSSP4* unexpectedly harboured expression in 3′ direction in endosperm. This exception was perhaps resulted from the interaction between expression regulatory sequences. Previous studies have shown that interactions between *cis*‐regulatory elements can produce different results than expected (Wang *et al*., 2015b). Hence in the present study, with the intercomparison among *BiGSSP2*,* BiGSSP4* and *BiGSSP7*, the alteration in expression pattern of *BiGSSP4* in endosperm could be attributed to the interaction between upstream reverse *OsTub6 intron* and *P*_*Osrbcs‐62*_, meanwhile, this interaction may be further amplified by *OsAct1 intron*. For another example, the degree of upregulation in *GUS* expression between *BiGSSP5* and *BiGSSP6* was overtly higher than that between *BiGSSP1* and *BiGSSP2*, which still further testify the diverse interactions between various expression regulatory sequences.

By comparing the *GFP* expression in *BiGSSP1* and *BiGSSP2*, we found that the upstream reversed *OsAct1 intron* can also be performed promoting effect. Moreover, the promoting intensity depended largely on the interval length between *OsAct1 intron* and core promoter. This bidirectional regulatory ability of *OsAct1 intron* is first discovered, which possesses an apparent advantage in providing more optimal designs for the construction of bidirectional synthetic promoters. Referring to the results of *BiGSSP4* and *BiGSSP7* (Figures 1 and 4), *OsTub6 intron* harboured the ability to greatly improve the expression efficiency of synthetic promoter without effect on expression pattern. However, although *OsAct1 intron* performed similar promoting effect as *OstTub6 intron*, the expression pattern altered to some extent. For example, the expression efficiency of *BiGSSP2*,* BiGSSP3* and *BiGSSP4* at 3′ direction is greatly promoted while leading to the change of expression pattern in green tissues compared to *BiGSSP1* (Figure 5). Meanwhile, both the addition of *OsAct1 intron* and *OsTub6 intron* showed significant promoting effects rather alter the green tissue specificity (except *BiGSSP4*).

The merits of synthetic promoter depend to a large extent on its application potential. Expression efficiency is one of the most important indicators. By comparison with the activity of *35S* promoter in green tissues, the expression efficiency towards 3′ direction overtly increased among *BiGSSP2*,* BiGSSP3*,* BiGSSP4*,* BiGSSP6* and *BiGSSP7*. The similar activity as *35S* towards 5′ direction was also found in *BiGSSP4* and *BiGSSP7*, while slightly lower expression efficiency occurred in *BiGSSP2* and *BiGSSP3*. As a result of the activity of *BiGSSP4* in endosperm, we can come to the conclusion that *BiGSSP7* ought to be the optimal bidirectional green tissue‐specific synthetic promoter constructed herein with strong expression efficient in both two directions. For all this, *BiGSSP2*,* BiGSSP3* and *BiGSSP6* also could be good candidates for application. Promoters with high expression in leaf (such as *BiGSSP7*) are suitable for transgenic breeding to improve disease and pest resistance in rice leaf (Chen *et al*., 2010a; Ye *et al*., 2010), as well as for the researches of photosynthesis (Cao *et al*., 2018), leaf senescence (Zhou *et al*., 2013) and so on. Meanwhile, promoters harboured high expression efficiency in stem can facilitate the future studies on stem elongation (Jiang *et al*., 2010) and the resistance to pest (Divya *et al*., 2018; Russell *et al*., 2018). In the process of the construction of synthetic promoters, various promoters can be constructed according to corresponding purposes and requirements for the customization of genetic engineering researches. Moreover, in the present study, the length of these four applicable synthetic promoters are all shorter than many native unidirectional tissue‐specific promoters, while the longest regulatory sequence (*BiGSSP7*) is 1896 bp, which shows remarkable convenience and operability for future application.

### Construction Strategy of bidirectional green tissue‐specific synthetic promoters

The application of native bidirectional promoter with high expression efficiency in plant was generally suffocated by its long sequence. Consequently, the synthetic promoters constructed by selective fusion of shorter expression regulatory sequences exhibiting similar expression efficiency can reduce complexity of synthesis and facilitated progress of application (Wang *et al*., 2015b). Simultaneously, the manner to promote synthetic promoter efficiency by fusing intron (*OsTub6I, OsAct1*) worked well without changing general expressional pattern. Furthermore, our work showed that the bidirectional synthetic promoters could be constructed by reusing the same core promoters in two reverse directions, and the expression could be enhanced by intron (*OsTub6I* and *OsAct1*). For instance, Huang (2006) reported that *P*_*Osrbcs‐62*_ showed high expression efficiency in leaf but no expression in stem. In our study, restoring expression efficiency of *BiGSSP1* in stem demonstrated that *P*_*Osrbcs‐62*_ and *P*_*Osrbcs‐550*_ could share regulatory elements in *P*_*Osrbcs‐550*_, which indicated that this manner to synthesize bidirectional promoters can shrink the construction cost and meet the demand of application.

### Regulatory sequences available for bidirectional tissue‐specific synthetic promoters

**Figure 6:**
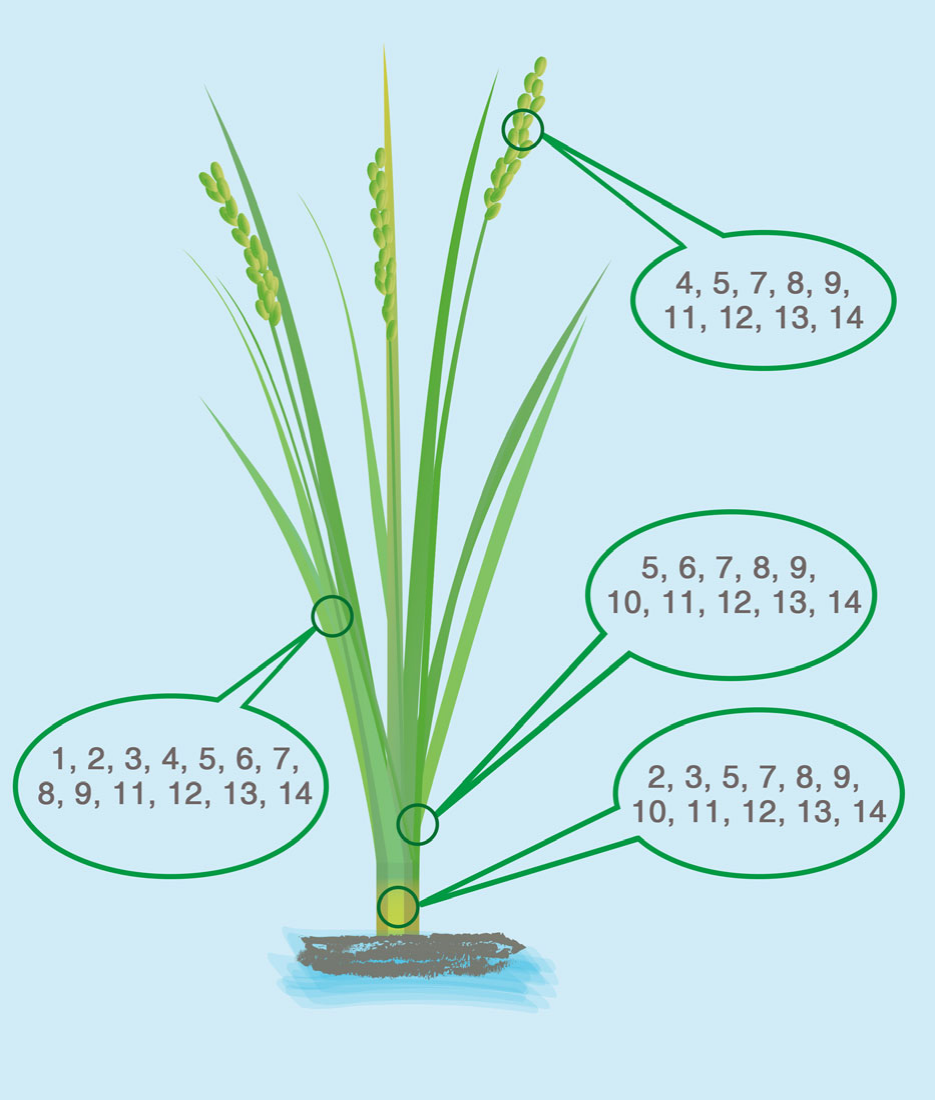
Schema of various green tissue‐specific expression regulatory sequences in rice. The code numbers of expression regulatory sequences expressed in corresponding tissues are showed in the box. 1, *Rca*; 2, *Ppask*; 3, *OrGSEp‐374*; 4, *LP2*; 5, *P*_*O*_ _*srbcs‐550*_; 6, *P*_*O*_ _*srbcs‐62*_; 7, *P*_*D*_ _*540‐544*_; 8, *P*_*DX*_ _*1*_; 9, G box; 10, GATA; 11, GEAT; 12, *OsAct1 intron*; and 13, *OsTub6 intron*.

Synthetic promoters will play an increasingly important role in rice genetic engineering due to their multifaceted application advantages. In particular, the research concerning gene expression in green tissues (such as photosynthesis‐related genes) plays a pivotal role in crop improvement and breeding. Given the fact that there are few studies on the tissue‐specific synthetic promoter in rice, let alone with bidirectional driving activities, we reviewed the green tissue‐specific expression regulatory sequences obtained in recent years as a repository available for future researchers (Figure 6). Considering that the length of regulatory sequence is closely related to the usability and flexibility of synthetic promoters, the length, specificity and expression efficiency of *cis*‐regulatory elements were taken into account (Cai *et al*., 2010; Huang, 2006; Lin *et al*., 2017; Thilmony *et al*., 2010; Xu *et al*., 2018; Yang *et al*., 2012). Furthermore, it should be paid particular attention that *cis*‐regulatory elements assembled in synthetic promoters always bring inconsistent function with earlier reports (Wang *et al*., 2015b). For example, our previous research has found the green tissue‐related *cis*‐regulatory element GEAT. However, it didn't show any promoting effect on the activity of *BiGSSP3* compared with *BiGSSP2*. It was inferred that the selection of *cis*‐regulatory elements should be based on the selected core promoter to avert unsatisfactory consequence. Therefore, in Figure 6, only those *cis*‐regulatory elements already used in synthetic promoters were depicted (Donald and Cashmore, 1990; Gianì *et al*., 2009; Puente *et al*., 1996; Wang *et al*., 2015b). Moreover, given that the improvement of other tissue or organ‐specific traits is of great significance in plant genetic engineering, we provide several classic flower, root and seed‐specific expression regulatory sequences in Table S4. These expression regulatory sequences combined with our construction strategy of synthetic promoters could provide the related researchers with an approach to construct other tissue‐specific synthetic promoters.

Our study first provided a feasible strategy for synthetic promoter construction and successfully constructed four bidirectional green tissue‐specific synthetic promoters. There synthetic promoters were also the first reported bidirectional green tissue‐specific promoters that showed high application potential in genetic engineering. Through the comparison among diverse designs of synthetic promoters in the present study, we analysed the regulatory role of various core promoters and *cis*‐regulatory elements towards different directions in synthetic promoters. *OsTub6 intron* and *OsAct1 intron* are proved as applicable candidates without abolition of green tissue specificity. By consideration of the expression efficiency in both directions, *BiGSSP7* is the first bidirectional green tissue‐specific synthetic promoter applicable for genetic engineering, which performed huge utilization potentiality. While some insufficiency still needed optimizing, such as the complex interactions between *cis*‐regulatory element and diverse promoters. In addition, since current research is limited in rice, the general applicability of this strategy for bidirectional promoter construction needs to be further explored. Given the few researches about green tissue‐specific promoter, mining more green tissue‐specific expression regulatory sequences alternative for synthetic promoters will become a big challenge in further future.

## Experimental procedures

### Scoring and selection of expression regulatory sequences

The application potential of synthetic promoters is closely related to their performance in tissue specificity, expression efficiency, sequence length and compatibility. However, it is almost impossible for a synthetic promoter to perform well in all aspects above. In the process of constructing synthetic promoters, these factors should be concerned with different degrees to meet individual demands. Consequently, we selected ten expression regulatory sequences based on a statistical results of relevant existing reports (Cai *et al*., 2010; Carlos *et al*., 2014; Gianì *et al*., 2009; Huang, 2006; Jeong and Jung, 2015; Kuwano *et al*., 2011; Lin *et al*., 2017; Lu *et al*., 2008; Manikandan *et al*., 2016; Molla *et al*., 2013; Nguyen *et al*., 2015; Park *et al*., 2015; Wang *et al*., 2015b; Xu *et al*., 2018; Yang *et al*., 2014; Zhang *et al*., 2014). The four features of each expression regulatory sequence (tissue specificity, expression efficiency, sequence length and universality) were, respectively, scored based on our scoring standard, as shown in Table S1 in detail. The total score of expression regulatory sequences were calculated by a weighted ratio, which is designed as follows:

Taking our goal to synthesize green tissue‐specific promoters into account, the tissue specificity of expression regulatory sequences is a crucial and conferred 45% weighted ratio. Besides, in view of the key role in determining the expression abundance of a target gene in applications, the expression efficiency is endowed 40% weighted ratio. Given the fact that short expression regulatory sequence could evidently reduce the cost of promoter construction, we grand 10% weighted ratio for regulatory sequence length. Considering original intention to meet application requirements, the universality and scalability of the expression regulatory sequences in different species should also be premeditated and awarded a weight of 5% (Table S2).

### Strategies for synthetic promoter assembling and scoring

From the perspective of gene co‐expression, we tend to construct the bidirectional promoters with the same expression pattern in two directions. One of the strategies for the construction of these promoters is to fuse a core sequence of a promoter to its upstream in reverse direction. This head‐to‐head arrangement allows the promoter to share regulatory sequence with the core sequence. Based on the conception above, we reversely appended *P*_*Osrbcs‐62*_, the core sequence of promoter *P*_*Osrbcs‐550*_, to the upstream of *P*_*Osrbcs‐550*_ to generate bidirectional promoter *BIGSSP1* (Figure 1). Due to *P*_*Osrbcs‐62*_ with no activity in the stem and low activity in other tissue, the first intron of *OsActin* (*OsAct1*) was selected to attach to 3′ terminus of *P*_*Osrbcs‐62*_ to estimate the effect of *OsAct1* on bidirectional activity of *BIGSSP2* (Figure 1). Our previous study demonstrated that four tandem GEAT (4×GEAT) appending to upstream of minimal *35S* promoter increased *GUS* expression in green tissue. Consequently, 4×GEAT was also placed upstream of *P*_*Osrbcs‐62*_ to investigate the effect of 4×GEAT on expression efficiency and specificity of bidirectional promoter *BIGSSP3*. Besides, in order to increase the expression efficiency of bidirectional promoter *BIGSSP4* in *P*_*Osrbcs‐550*_ direction, the leader intron of *OsTubulin6* (*OsTub6I*) was selected as a regulatory sequence and located downstream of *P*_*Osrbcs‐550*_.

As far as crop genetic improvement is concerned, bidirectional promoters with the different expression patterns exhibit better application prospect. Therefore, the synthetic promoter *BIGSSP5* was conducted by a head‐to‐head combination of *P*_*Osrbcs‐550*_ and *P*_*D54O‐544*_ which provides an alternative to *P*_*Osrbcs‐62*_ in *BIGSSP1*. Simultaneously, *OsAct1* and *OsTub6I* were, respectively, applied to *BIGSSP6* and *BIGSSP7* to enhance their expression efficiency and green tissue specificity.

Based on above strategies, seven bidirectional green tissue‐specific promoters were designed with their schemes shown in Figure 1a and scored based on the type, number, length and location of expression regulatory sequences (Figure 1b).

### Sequence synthesis and vector construction

The sequences of seven bidirectional green tissue‐specific promoters were synthesized. All the constructs synthesized were ligated in promoter functional analysis vector pDX2181 after digesting with *Hind* III and *Pst* I to drive the reporter gene *GFP* and *GUS* expression.

### *Agrobacterium*‐mediated transformation to rice callus

All the constructed vectors and *pDX2181* as well as CaMV *35S‐pDX2181* were transformed into *Agrobacterium tumefaciens* strain *EHA105* by electroporation following a sequence confirmation, which subsequently was introduced into Zhonghua11 (*Oryza sativa* L. ssp. *japonica*) by Agrobacterium‐mediated transformation. The callus culture and transformation procedures were carried out as previously described (Hiei *et al*., 2010). The transgenic rice containing of *pDX2181* and CaMV *35S‐pDX2181* vector, respectively, serve as the negative control and positive control.

### Histochemical and fluorometric detection of GUS activity

Histochemical assay of GUS activity in transgenic rice was performed as described previously with follow modification (Jefferson *et al*., 1987). Various tissues of T_0_ transgenic positive transformants (root, leaf, sheath, panicle, stem and mature seed) were incubated in GUS staining solution (50 mm sodium phosphate at pH 7.0, 10 mm Na2‐EDTA, 0.1% Triton X‐100, 1 mg/mL X‐Gluc, 100 μg/mL chloramphenicol, 1 mm potassium ferricyanide, 1 mm potassium ferrocyanide and 20% methanol) at 37 °C for 12 h after 15‐min vacuum filtration. After GUS staining, samples were bleached with 70% (v/v) ethanol and viewed under a microscope (Leica MZFLIII).

Quantitative analyses of GUS activity in transgenic rice were conducted based on previous report (Xu *et al*., 2010). The protein concentrations were quantified as described by Bradford (1976). GUS protein in the supernatant was determined fluorometrically with an INFINITE 200 photometer (Tecan Austria GmbH, Ltd, Grödig, Austria). GUS activities were determined by measuring the amounts of 4‐Methylumbelliferone (4‐Mu) produced under the catalysis of GUS in 1 mg of total protein per minute.

### Histological and quantitative analysis of GFP

Histological analysis of GFP in rice tissues was conducted and photographed under fluorescence microscope. Various tissues of T_0_ transgenic positive transgene rice (root, leaf, sheath, panicle, stem and mature seed) were sampled and observed under a fluorescence microscope (Leica MZ16F) using GFP filter sets and Leica Application Suite software.

The relative expression levels of *GFP* in rice tissues were detected by quantitative real‐time PCR (qRT‐PCR). Total RNAs of different rice tissues were extracted and reverse‐transcribed as described previously (Wang *et al*., 2015a). Subsequently, a qRT‐PCR assay was performed by using GFP primers (GFP‐F: 5′‐ATCCGCCACAACATCGAGGA‐3′ and GFP‐R: 5′‐TCGTCCATGCCGAGAGTGAT‐3′). The *GAPDH* gene was selected as endogenous control with primers (GAPDH‐F: 5′‐CT GCAACTCAGAAGACCGTTG‐3′ and GAPDH‐R: 5′‐CCTGTTGTCACCCTGGAAGTC‐3′). Relative expression levels were determined using 2^−△△C^_T_ method (Yu *et al*., 2007) and normalized by the expression level *GFP* driven by *RB* in stem.

## Author contributions

Rui Wang involved in conceptualization and funding acquisition; Jiuyuan Bai, Xin Wang and Fei Ling involved in investigation; Xin Wang, Jiuyuan Bai and Hao Wu involved in data analysis and visualization; Jiuyuan Bai and Xin Wang involved in original draft writing; Yun Zhao and Yongjun Lin involved in writing review and editing.

## Supporting information


**Table S1** The scoring standard of expression regulatory sequence.

**Table S2** The scoring and screening of expression regulatory sequence. The score of each item ranges from 0 to 10.

**Table S3** The information of expression regulatory sequences used in our study.

**Table S4** Other tissue‐specific expression regulatory sequences.
