# Supplementary material for: Comprehensive construction strategy of bidirectional green tissue‐specific synthetic promoters
Source: Plant Biotechnol J. 2019 Aug 19;18(3):668–78. doi: 10.1111/pbi.13231 (PMC7004895; doi:10.1111/pbi.13231)
Supplement: Supplementary file 1 — Table S1 The scoring standard of expression regulatory sequence. [file PBI-18-668-s003.docx]

**Table S1 The scoring standard of expression regulatory sequences**

| Scoring standard of specificity | | Scoring standard of expression activity | | Scoring standard of sequence length score | | Scoring standard of universality | |
| --- | --- | --- | --- | --- | --- | --- | --- |
| Expression pattern | Score range | Relative expression activity compared to CaMV *35S* | Score range | Length | Score range | Application range in species | Score range |
| No expression in green tissues | 0 | 0%-20% | [0, 2] | 1-100bp | [8, 10] | 1 specie | [1,3] |
|  |  | 20%-40% | [2, 4] | 100-300bp | [6, 8] | 2 species | (3,5] |
| Expressing in green tissues and also in non-green tissues | (0, 5] | 40%-60% | [4, 6] | 300-600bp | [4, 6] | 3 species | (5,7] |
|  |  | 60%-80% | [6, 8] | 600-1000bp | [2, 4] | 4 species | (7,9] |
| Expressing only in green tissues | (5, 10] | 80%-100% | [8, 10] | 1000-2000bp | [0, 2] | More than 4 species | (8,10] |
