# Supplementary material for: Comprehensive construction strategy of bidirectional green tissue‐specific synthetic promoters
Source: Plant Biotechnol J. 2019 Aug 19;18(3):668–78. doi: 10.1111/pbi.13231 (PMC7004895; doi:10.1111/pbi.13231)
Supplement: Supplementary file 2 — Table S2 The scoring and screening of expression regulatory sequence. The score of each item ranges from 0 to 10. [file PBI-18-668-s002.docx]

**Table S2 The scoring and screening of expression regulatory sequences**

| Regulatory sequences name | Expression specificity score | Expression activity score | Sequence length score | universality score | total weighted score |
| --- | --- | --- | --- | --- | --- |
| GT1 | 1 | 1 | 8 | 6 | 1.95 |
| GEAT | 2 | 1 | 8 | 2 | 2.2 |
| *OrGSEp-374* | 4 | 4 | 6 | 2 | 4.1 |
| *OsAct1 intron* | 4 | 4 | 6 | 5 | 4.25 |
| *OsTub*6 *intron* | 1 | 4 | 6 | 4 | 2.85 |
| P_D540_ | 5 | 5 | 3 | 7 | 4.9 |
| P_D540-544_ | 8 | 8 | 6 | 8 | 7.8 |
| *P_Osrbcs-62_* | 8 | 3 | 10 | 6 | 6.1 |
| *P_Osrbcs-550_* | 7 | 7 | 6 | 6 | 6.85 |
| *P_Psak_* | 7 | 5 | 5 | 2 | 5.75 |

The score of each item ranges from 0 to 10.
