# Supplementary material for: Comprehensive construction strategy of bidirectional green tissue‐specific synthetic promoters
Source: Plant Biotechnol J. 2019 Aug 19;18(3):668–78. doi: 10.1111/pbi.13231 (PMC7004895; doi:10.1111/pbi.13231)
Supplement: Supplementary file 3 — Table S3 The information of expression regulatory sequences used in our study. [file PBI-18-668-s004.docx]

**Table S3 the information of expression regulatory sequences used in our study**

| Regulatory sequences name | Sequences |
| --- | --- |
| GEAT | ATATT |
| *OsAct1 intron* | GTAACCACCCCGCCCCTCTCCTCTTTCTTTCTCCGTTTTTTTTTTCGTCTCGGTCTCGATCTTTGGCCTTGGTAGTTTGGGTGGGCGAGAGCGGCTTCGTCGCCCAGATCGGTGCGCGGGAGGGGCGGGATCTCGCGGCTGGCGTCTCCGGGCGTGAGTCGGCCCGGATCCTCGCGGGGAATGGGGCTCTCGGATGTAGATCTTCTTTCTTTCTTCTTTTTGTGGTAGAATTTGAATCCCTCAGCATTGTTCATCGGTAGTTTTTCTTTTCATGATTTGTGACAAATGCAGCCTCGTGCGGAGCTTTTTTGTA |
| *OsTub*6 *intron* | GTGAATTCCGCAACAAAAAATCTTTCGATTCGCTTCCCCCCGAATTATCTAGCGCCTCGTGCTTTCCGGTAGAATTTTTCTTTGGGTCCGATCGATTCTTTTCTTTTTTTTTTGGGGGGGGGTTGGGGGTGGGTGGATTATTGAGGATTTGAGGTGTTCTTGGTGCGATCGGAACATTTTTGGTTCGTTTGTTTGGGGATTCTTGCGGTGTTCTGGGTAGAATCAGGACTAGCTGTTCTTCTCGGTGTTCCGATCGTTTGGGGATTAGTGAGGATTTGAGGTGTTCTTGGTGCGATCAGAAGATTTTTGGTTCGTTTTTTTTCTCTTTCGGATTCTTGCGGTGTTCTTGGTAGAATCAGTACTCTCGGTGTTCCGATTCCCGCGTTGGAATTTCAGAAGGTCTCACCCACCCTGACGTTTCGCGTTTCGTGGCGATTTCTCTGCAG |
| P_D540-544_ | TAGATAATGGATTAAAACCTGGCCTCTATATCCAAACTCGGATGTACATGGCCAAATTAACATCCAGTTCTTCATCCACCTTCATATCAGAACATTCCAGAATACAGATTGCAATGGCATCCATTGGCACCACATGTTTTGACTGATTCTTTCACTGTTTCTTGATATATTTGTTGATTTTTAATGGTAAATAGCTCATCTGTCCAATCCTGGCACCACATAATTTCCCCTGTTAAAACAGTGCCAACACAAATTTACTCATGTCCACTGAAAATCCCCAGAGGATAACGTTCTGGCACTGCCATATCAGGACCGGACCATACGCGGCGCGCCACGTGGCGCGCCGGCGGTCACACCTGTGCTGGTGCACCCTGGCATTATCCCCATCCACCATCCTGTGGGTGACTGGCCACCAAAGGTTTTTGGCGCCACAATTATATTGAGCTGCCATTGCTTCTCACCTCTGCTTTGCATCCATCCATCCATCAGAGATCAGGTAGAACAGTGAGAGTGAGGTGCAGAAAAATTTGAGCTGAAGCTGAGGATGGCAACATCCATGATCACCTCGCCGCTGGTGGCGCCGGC |
| *P_Osrbcs-62_* | GATCCGCTGAGTTTTGGCTATTTATACGTACCGCGGGAGCCTGTGTGCAGAGCAGTGCATCT |
| *P_Osrbcs-550_* | GCTTAATGAGGGCCCAAAGTTTTGATGACCTTTTGCTTGATCTCGAAATTAAAATTCAAGTACCTGTTAAGGGAGGTCACACCACCATCAATTTTCAGCCTGAAGAAACAGTTAAACAACGACCCCGATGACCAGTCTACTGCTCTCCACATACTAGCAGCATTATTGATCACAAAAGAAACCAAAATAAAAATCAGCACCGAGTGTGCAGAGGGAGACAAAGGTGATCTGGCAGTGGATATCTCCCCATCCATCCTCACCCGCGCTGCCCATCACTCGCCGCCGCATACTACATCATGTGGAGAGAGGAAGACGAGGACCACAGCCAGAGCCCGGGTCGAGATGCCACCACGGCCACAATCCACGAGCCCGGCGCGACACCACCGCGCGCGCGTGAGCCAGCCACAAACGCCCGCGGATAGGCGCGCGCACGCCGGCCAATCCTACCACATCCCCGGCCTCCGCGGCTCGCGAGCGCCGCTGCCATCCGATCCGCTGAGTTTTGGCTATTTATACGTACCGCGGGAGCCTGTGTGCAGAGCAGTGCATCT |
