# Supplementary material for: Comprehensive construction strategy of bidirectional green tissue‐specific synthetic promoters
Source: Plant Biotechnol J. 2019 Aug 19;18(3):668–78. doi: 10.1111/pbi.13231 (PMC7004895; doi:10.1111/pbi.13231)
Supplement: Supplementary file 4 — Table S4 Other tissue‐specific expression regulatory sequences. [file PBI-18-668-s001.docx]

**Table S4 Other tissue-specific expression regulatory sequences**

| tissue specificity | Regulatory sequences name | Gene name | References |
| --- | --- | --- | --- |
| Flower specificity | CArG boxes | PsEND1, | Gómez et al. (2004) |
|  | LAT52 | LAT | Albani et al. (1991) |
|  | *pFKP1* | *FKP1* | Ishiguro et al. (2010) |
|  | *pdAGP3* | MdAGP3 | Choi et al. (2011) |
|  | OSIPA199 | OSIPA | Swapna et al. (2011) |
| Root specificity | *ROOTMOTIFFTAPOX1* | *rolD* | Elmayan and Tepfer (1995) |
|  | *RSE* | *Glycine-rich protein 1.8* | Beat and Christine (1991) |
|  | *rolD* | *rolD* | Leach and Aoyagi (1991) |
|  | *P1107* | *Os03g01700* | Li et al. (2019) |
|  | *RHERPATEXPA7* | *EXPA7* | Kim et al. (2006) |
| Seed specificity | RY repeat | *OsRAB16, ZmRAB17,* | Baumlein (2010) |
|  | *GCN4* | *OsGluB-1* | Wu et al. (1998) |
|  | *Skn-1* | *OsGluB-1* | Washida et al. (1999) |
|  | Prolamin box | *ZmZ4, ZmZ19, ZmZ27* | Ueda et al. (1994) |
|  | *GLYCINE-RICH PROTEIN 7* | *OsGRP7* | Xue et al. (2016) |

**References**

Albani D, Altosaar I, Arnison P, G., et al. (1991) A gene showing sequence similarity to pectin esterase is specifically expressed in developing pollen ofBrassica napus. Sequences in its 5′ flanking region are conserved in other pollen-specific promoters. *Plant Molecular Biology*. 16:501-513.

Baumlein H (2010) *Cis*-analysis of a seed protein gene promoter : the conservative RY repeat CATGCATG within the legumin box is essential for tissue specific expression of a legumin gene. *Plant Journal for Cell & Molecular Biology*. 2:233-239.

Beat K, Christine B (1991) Vascular-Specific Expression of the Bean *GRP 1.8* Gene Is Negatively Regulated. *Plant Cell*. 3:1051-1061.

Choi H, Jin JY, Choi S et al. (2011) An ABCG/WBC‐type ABC transporter is essential for transport of sporopollenin precursors for exine formation in developing pollen. *Plant Journal*. 65:181-193.

Elmayan T, Tepfer M (1995) Evaluation in tobacco of the organ specificity and strength of therolD promoter, domain A of the *35S* promoter and the 35S2 promoter. *Transgenic Research*. 4:388-396.

Gómez MD, Beltrán J, Luis ACa (2004) The peaEND1promoter drives anther-specific gene expression in different plant species. *Planta*. 219:967-981.

Ishiguro S, Nishimori Y, Yamada M et al. (2010) The Arabidopsis *FLAKY POLLEN1* Gene Encodes a 3-Hydroxy-3-Methylglutaryl-Coenzyme A Synthase Required for Development of Tapetum-Specific Organelles and Fertility of Pollen Grains. *Plant & Cell Physiology*. 51:896-911.

Kim DW, Lee SH, Choi SB et al. (2006) Functional Conservation of a Root Hair Cell-Specific *cis*-Element in Angiosperms with Different Root Hair Distribution Patterns. *Plant Cell*. 18:2958-2970.

Leach F, Aoyagi K (1991) Promoter analysis of the highly expressed *rolC* and *rolD* root-inducing genes of Agrobacterium rhizogenes: Enhancer and tissue-specific DNA determinants are dissociated. *Plant Science*. 79:69-76.

Li Y, Li C, Cheng L et al. (2019) Over-expression of OsPT2 under a rice root specific promoter Os03g01700. *Plant Physiol Biochem*. 136:52-57.

Swapna L, Khurana R, Kumar SV et al. (2011) Pollen-Specific Expression ofOryza sativa IndicaPollen Allergen Gene (*OSIPA*) Promoter in Rice andArabidopsisTransgenic Systems. *Molecular Biotechnology*. 48:49-59.

Ueda T, Wang Z, Pham N et al. (1994) Identification of a transcriptional activator-binding element in the 27-kilodalton zein promoter, the -300 element. *Molecular & Cellular Biology*. 14:4350-4359.

Washida H, Wu C, Akihiro S et al. (1999) Identification of *cis*-regulatory elements required for endosperm expression of the rice storage protein glutelin gene *GluB-1*. *Plant Molecular Biology*. 40:1-12.

Wu CY, Adach T, Hatano T et al. (1998) Promoters of Rice Seed Storage Protein Genes Direct Endosperm-Specific Gene Expression in Transgenic Rice. *Plant & Cell Physiology*. 39:885-889.

Xue GP, Rae AL, White RG et al. (2016) A strong root-specific expression system for stable transgene expression in bread wheat. *Plant Cell Rep*. 35:469-481.
